# Health related quality of life improvement in chronic non-specific neck pain: secondary analysis from a single blinded, randomized clinical trial

**DOI:** 10.1186/s12955-018-1032-6

**Published:** 2018-11-06

**Authors:** Ester Cerezo-Téllez, María Torres-Lacomba, Orlando Mayoral-del-Moral, Soraya Pacheco-da-Costa, David Prieto-Merino, Beatriz Sánchez-Sánchez

**Affiliations:** 1 Alcalá de Henares University, Physiotherapy in Women’s Health Research Group Department of Physiotherapy, Physiotherapy Faculty, University of Alcalá, Carretera Madrid – Barcelona, km 33,600, E-28871 Alcalá de Henares, Madrid, Spain; 2 Primary Health Care, Alcalá de Henares, Madrid, Spain; 3 Physical Therapy Unit, Provincial Hospital, Toledo, Spain; 4 Physical Therapy, Disability and Dependence Research Group, Physical Therapy Department, University of Alcalá, Alcalá de Henares, Madrid, Spain; 5 Applied Statistical Methods in Medical Research Group, Catholic University of Murcia, UCAM, Murcia, Spain

**Keywords:** Health-related quality of life, Neck pain, Myofascial pain syndrome, Myofascial trigger points, Physical therapy, Deep dry needling, Muscle stretching techniques, Primary care, Randomized clinical trial

## Abstract

**Background:**

Chronic non-specific neck pain is related to limited cervical mobility, impaired function, neck muscles myofascial pain syndrome, and stress at work. The aforementioned factors are strongly related and may lead to a negative impact on health-related quality of life. There are some effective conservative Physical therapy interventions for treating chronic non-specific neck pain. Currently, Deep Dry Needling is emerging as an alternative for improving symptoms and consequently, the quality of life in patients with chronic non-specific neck pain. The purpose of the study was to examine the effectiveness of Deep Dry Needling of myofascial trigger points on health-related quality of life improvement, as a secondary analysis, in people with chronic non-specific neck pain.

**Methods:**

A randomized parallel-group blinded controlled clinical trial was conducted at a public Primary Health Care Centre in Madrid, Spain, from January 2011 to September 2014. One hundred thirty subjects with chronic non-specific neck pain and active myofascial trigger points in neck muscles were randomly allocated into two groups. Subjects in the intervention group (*n* = 65) were treated with Deep Dry Needling in active myofascial trigger points plus stretching in neck muscles; Control group (*n* = 65) received only stretching. Both interventions lasted 2 weeks, 2 sessions per week. Health-related quality of life was measured with Short Form-36 (SF-36), in 5 assessments: at baseline, after intervention period; and at 1, 3 and 6 months after intervention.

**Results:**

For both groups, SF-36 mean values increased in all dimensions in every assessment. Significant differences (*p* < 0.05) were found in favor of the intervention group for all dimensions at the last assessment. For some dimensions (physical function, physical role, social function and vitality), the evidence was more consistent from the beginning.

**Conclusions:**

Deep Dry Needling plus stretching is more effective than stretching alone for Health-related quality of life improvement, especially for physical function, physical role, social function and vitality dimensions, in people with non-specific neck pain.

**Trial registration:**

Current Controlled Trials ISRCTN22726482. Registered 9 October 2011.

## Background

Up to 67% of world’s population may present chronic non-specific neck pain at least once in their lives. There is a relationship between functional limitation and disability in individuals with chronic pain, and they use health services and medication for pain relief very often. It is considered a public health and it is a frequent cause of job absenteeism which provokes high socioeconomic costs [1–3].

Chronic non-specific neck pain is diagnosed as cervical pain without a known pathological basis as the underlying cause of the complaints. Some symptoms are limited cervical spine mobility and neck muscles weakness, which may be often related to other problems, such as, vertebral, neck or shoulder impaired function, and mental and physical stress at work. Besides, chronic non-specific neck pain patients have more functional limitations and catastrophizing beliefs that may cause disability, lower vitality and worse general health status. All the aforementioned factors are strongly related, affect one into the other, and may lead to a negative impact on health-related quality of life (HRQoL) [2, 4–6].

There are different treatments for patients with chronic non-specific neck pain and many of them describe the need of a multidisciplinary approach [7–12]. Regarding physical therapy interventions for treating chronic non-specific neck pain symptoms, the most common treatments include exercise therapy [7, 13], stretching [8, 9, 14], electrotherapy [15] and manual therapy [7, 10].

Some recent studies have also reported the relation between chronic non-specific neck pain and Myofascial Pain Syndrome (MPS), caused by myofascial trigger points (MTrPs) in cervical muscles with a high prevalence in trapezius, levator scapulae, multifidi cervicali and splenius cervicis muscles [5]. The most frequent conservative physical therapy interventions for treating MPS are stretching, massage, ischemic compression, and pressure release techniques [4, 16–19]. The effectiveness of Deep Dry Needling (DDN), an invasive technique which is included in some physical therapy interventions for treating MPS, has also been reported, in different studies, to improve pain intensity, mechanical hyperalgesia, neck range of motion, neck muscle strength and neck disability [4, 20–24]. The benefits of DDN for the aforementioned symptoms have been described in the primary analysis of this study and the results showed better and clinically meaningful results for all of them, when compared with control group in the short-term and at 6-month follow-up [2, 4–6].

There are some studies that report HRQoL in patients with chronic non-specific neck pain about different physical therapy interventions, such as global posture reeducation and static stretching [8]; neck strength training [25]; physical training, specific exercises and pain education [26]; and home-based exercise [27]. However, as far as the authors know, no studies relate chronic non-specific neck pain, MPS, DDN of MTrPs and HRQoL.

Therefore, in the present study, a secondary analysis was performed in order to determine HRQoL improvement, providing new data not described in the primary analysis [2, 4–6].

## Methods

### Aim

To determine the effectiveness of DDN of MTrPs on HRQoL improvement in people with chronic non-specific neck pain [2, 4–6].

### Design and setting

This paper reports a secondary analysis of the study *“Effectiveness of dry needling in chronic non-specific neck pain: randomized, single blinded, clinical trial”* which was carried out between January 2011 and September 2014 at a Primary Health Care Center in Alcalá de Henares (Madrid- Spain)X by the Physiotherapy in Women's Health Research Group. It was approved by the Human Ethics Committee at Principe de Asturias Hospital in Alcalá de Henares, Madrid (Spain) [4].

### Participants

The sample was recruited at 3 primary health care centers in Alcalá de Henares (Madrid) and consisted of 130 participants who gave written informed consent to participate in the study. All participants were diagnosed with chronic non-specific neck pain by their primary care doctor [4, 28].

After the diagnosis, a trained physical therapist, with more than 15 years of experience in the diagnosis and treatment of MTrPs, assessed each participant with a standardized clinical physical therapy assessment of the neck and upper extremities to determine if there was MPS in neck muscles. Those subjects who presented at least 1 active MTrP in elevator scapulae, trapezius, multifidi or splenius cervicis muscles, according to the diagnosis criteria established by Simons et al. [29], were included in the study. The assessment was performed by a group-affiliation-blinded expert physical therapist with more than 10 years of experience on assessing and treating MPS [4].

After signing the informed consent, participants were randomly allocated into 2 groups: DDN group (DDN; *n* = 65) and control group (CG; *n* = 65). Sample size was calculated according to the main objective of the original clinical trial.

Details on sample size, sample recruitment, randomization, and blinding are explained in the paper with the primary analysis by Cerezo et al. [4].

### Interventions

Physical therapy interventions in both groups consisted on 20-min sessions, twice a week, during 2 weeks, and were carried out by 2 experienced physical therapists with more than 10 years of experience in the treatment of MTrPs at a primary health care center in Alcalá de Henares, Madrid.

DDN group intervention was performed by physical therapist 1 (Pt1) and included DDN for each active MTrP found in multifidi cervicis, esplenius cervicis, levator scapulae and trapezius muscles using a 4 cm × 0.32 mm acupuncture needle with a guided tube (ASP. A1040P. Agu-punt S.L. acupuncture-Physiotherapy. Barcelona, Spain). After DDN, a passive stretching of splenius cervicis, cervical multifidi, levator scapulae and trapezius muscles was performed 4 times in the positions described by Simons et al. [29]. In the CG, physical therapist 2 (Pt2) performed the same passive stretching of the above mentioned muscles.

Before the study started, a series of consensus meetings were carried out in order to ensure both physical therapists (Pt1 & Pt2) would perform the same passive stretching intervention. They were the only study members aware of group allocation.

During the intervention period, if participants referred high pain intensity, they were treated with the rescue medication proposed by their primary care physician. No participant was treated out of the interventions established in the study.

### Outcome assessments

Patients were assessed 5 times: at baseline (A0), just after the intervention period (A1–3 weeks from baseline), and then at 1 month (A2–7 weeks from baseline), 3 months (A3–16 weeks from baseline), and 6 months (A4–30 weeks from baseline) after the intervention. HRQoL was measured with the Short Form 36 Health Survey Spanish version 2 (SF-36v2) at each of these time points.

SF-36v2 is a generic instrument used to assess multidimensional HRQoL, which consists of 36 items encompassed in 8 dimensions: Physical Function (PF), Physical Role (PR), Bodily Pain (BP); General Health (GH), Vitality (VT), Social Function (SF), Emotional Role (ER) and Mental Health (MH) and 2 summary values for Physical Component Summary (SF-36 PSC) and Mental Component Summary (SF-36 MSC) [30]. Each dimension ranges from 0 (worst possible HRQoL) to 100 (best possible HRQoL).

### Statistical analysis

Participants’ characteristics and relevant health variables and HRQoL were compared between the two groups at baseline with descriptive statistics. To estimate the effect of the intervention on HRQoL over time, a separate baseline-adjusted linear regression model was used for each SF-36v2 dimension at each visit. The differences in SF-36v2 score from baseline to the visit was regressed over a binary variable for trial arm (0 = control, 1 = intervention) and the baseline values of the SF-36v2 dimension centered on the mean. This allows us to estimate the difference between the two trial arms of the changes from baseline visit while considering the possible “regression to the mean” that might typically occur when using repeated measures of the same variable. No correction was applied for multiple testing as many of these *p*-values are clearly not independent and no decision has to be taken based on these *p*-values. Actual *p*-values and confidence intervals are shown and the evidence for each outcome is discussed. The software R v.3 © was used for data analysis.

## Results

**Fig. 1:**
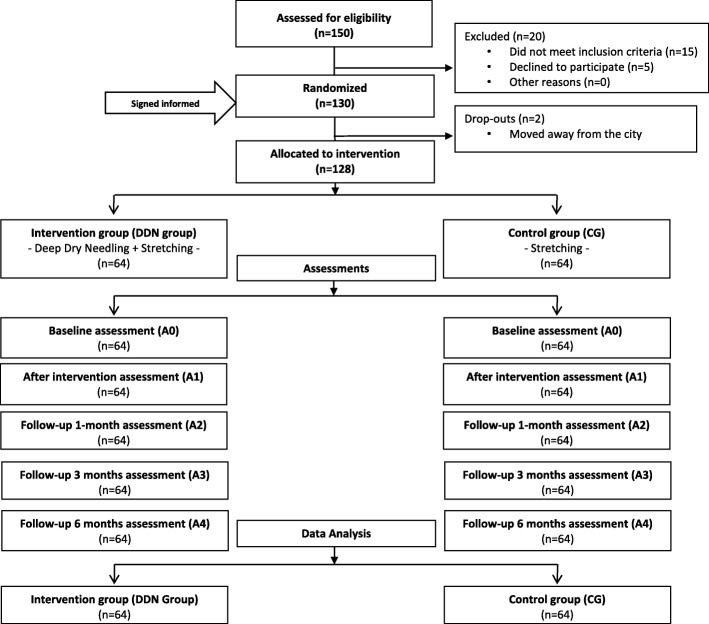
Flow diagram of participants throughout the study

Between January 2010 and December 2014, 150 subjects were recruited to participate in the study, as they were diagnosed with chronic non-specific neck pain by their primary care doctor. After excluding 20 subjects for not accomplishing inclusion criteria, 130 participants with chronic non-specific neck pain and active MTrPs in neck muscles were included in the study to receive physical therapy treatment. After randomization, 2 subjects dropped out because they moved away from the city. Therefore, 128 participants self-fulfilled SF-36 and more than 98% of the items were answered (Fig. 1). Although the sample and methodology was the same as the primary study, the results presented in this manuscript correspond to a new and secondary analysis of in order to analyze HRQoL with every SF-36v2 dimension.

Baseline demographics and descriptive pre-intervention statistics of the sample are shown in Table 1, according to the intervention groups. Both groups were fairly homogeneous at baseline, except for sex, with more females in the CG (45) than in DDN group (36); and BP dimension which had a lower value in CG (45.1) than in DDN group (59.6). As this is a randomized design, the main analysis will be uncontrolled by sex but adjusted by baseline HRQoL dimension to account for regression to the mean effect. However, a sensitivity analysis controlling for sex was done and the results were basically the same in all the outcomes (results not shown).

**Table 1:** Descriptive characteristics of the sample according to the intervention groups

Variable	Control Group (*N* = 64)	Deep dry needling group (*N* = 64)
	Mean (SD) or N (%)	Mean (SD) or N (%)
Age	52 (16.6)	48 (15.7)
Sex = female	45 (70.3%)	36 (56.3%)
Working status	53 (83%)	55 (86%)
Osteopenia	5 (8%)	5 (8%)
Osteoporosis	2 (3.1%)	1 (1.6%)
Arthrosis	14 (21.9%)	13 (20.3%)
Body mass index	26.3 (4.3)	26.2 (4.6)
HRQoL (SF-36)		
Physical Function	72.3 (19.6)	79.2 (14.5)
Physical Role	12.9 (11.7)	11.1 (11.2)
Bodily Pain	45.1 (22.5)	59.6 (24.2)
General Health	56(16.4)	59(15.7)
Vitality	54.1(20.8)	56.7(19.8)
Social Function	82.2(16.6)	83.0(15.5)
Emotional Role	16.9(11.6)	19.0(9.2)
Mental Health	69.1(14.9)	64.6(17.8)
SF-36 PSC	40.9(8)	43.8(4.1)
SF-36 MSC	40.8(6.6)	38.8(7.3)

*SD* standard deviation, *HRQoL* Health-Related Quality of Life, *SF-36 PSC* Physical Component Summary, *SF-36 MSC* Mental Component Summary

**Fig. 2:**
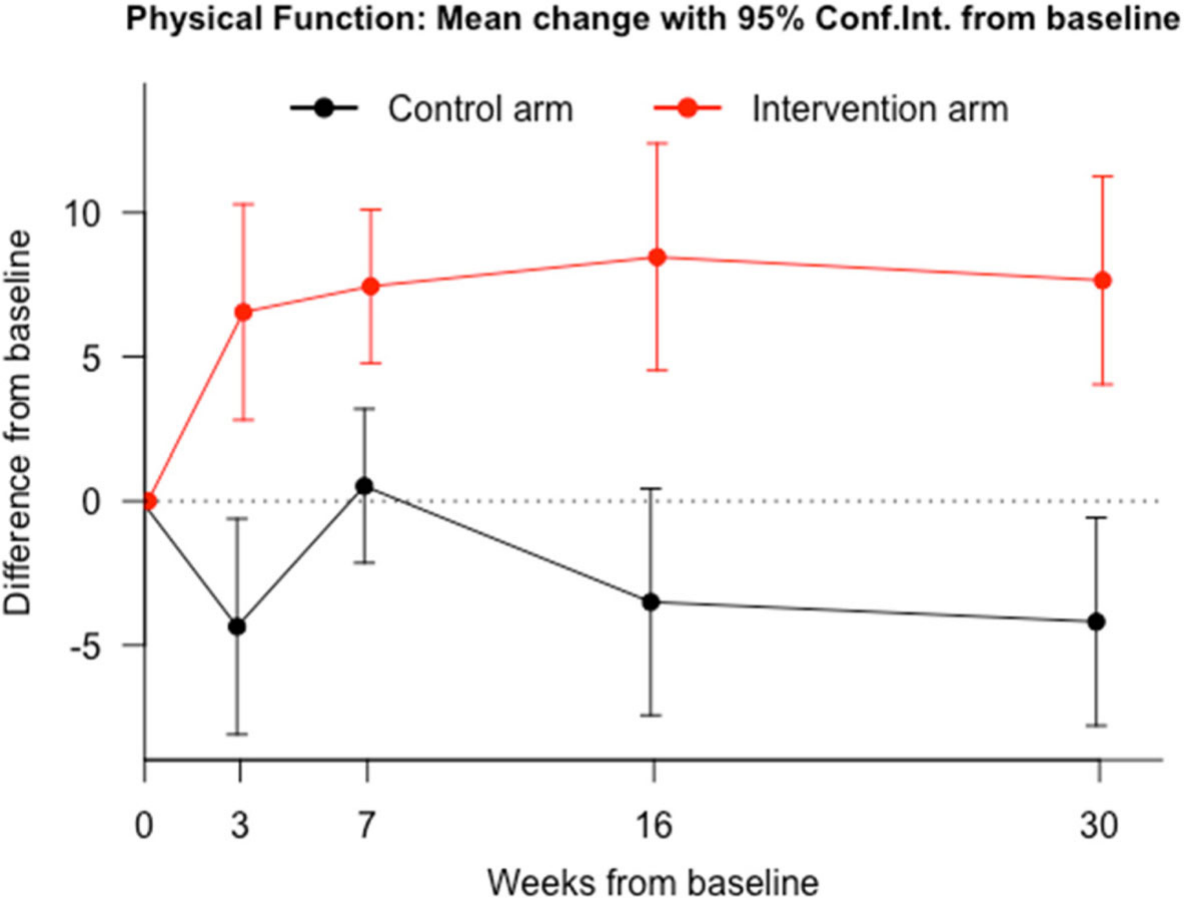
Evolution of physical function throughout the study in both groups. Comparison of means at baseline (A0), post-treatment at 3 weeks after baseline (A1), at 30 days follow-up after A1 (A2), at 3 months follow-up (A3), and at 6 months follow-up (A4)

**Fig. 3:**
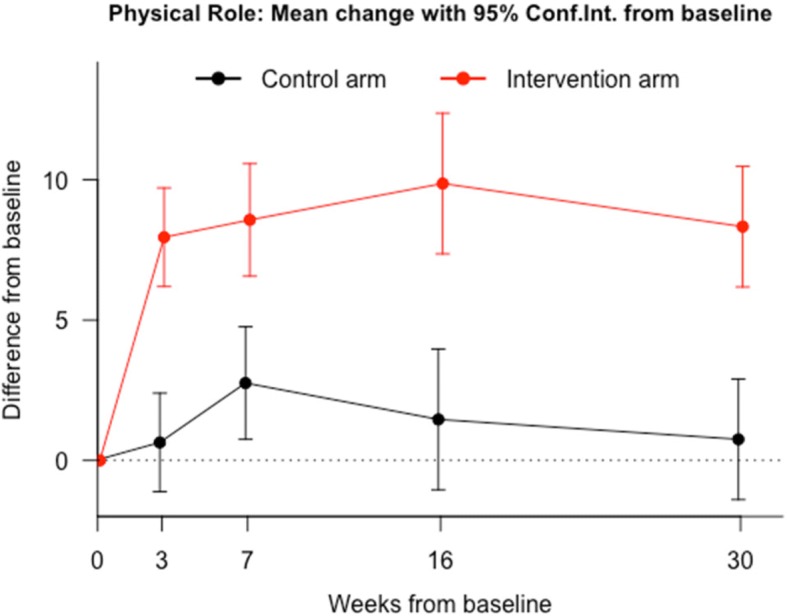
Evolution of physical-role throughout the study in both groups. Comparison of means at baseline (A0), post-treatment at 3 weeks after baseline (A1), at 30 days follow-up after A1 (A2), at 3 months follow-up (A3), and at 6 months follow-up (A4)

**Fig. 4:**
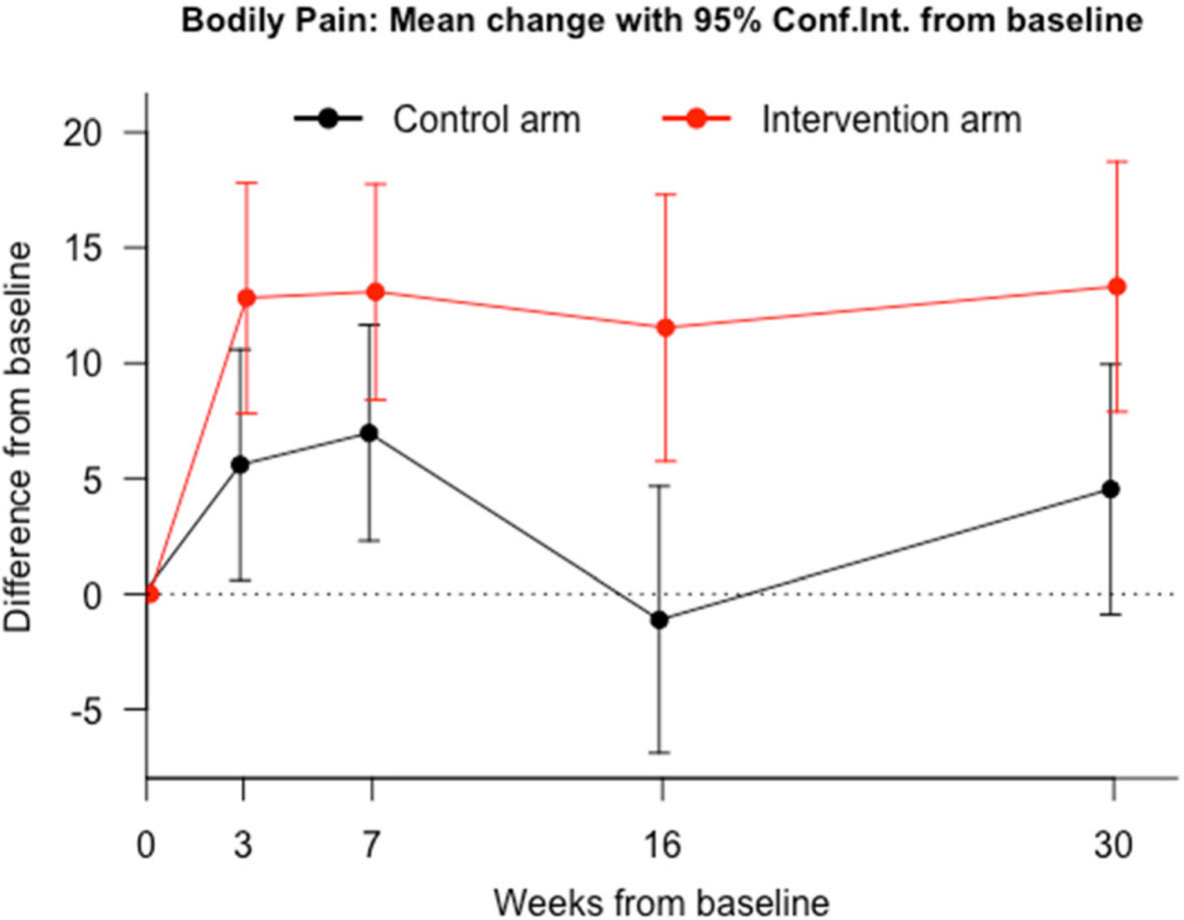
Evolution of bodily pain throughout the study in both groups. Comparison of means at baseline (A0), post-treatment at 3 weeks after baseline (A1), at 30 days follow-up after A1 (A2), at 3 months follow-up (A3), and at 6 months follow-up (A4)

**Fig. 5:**
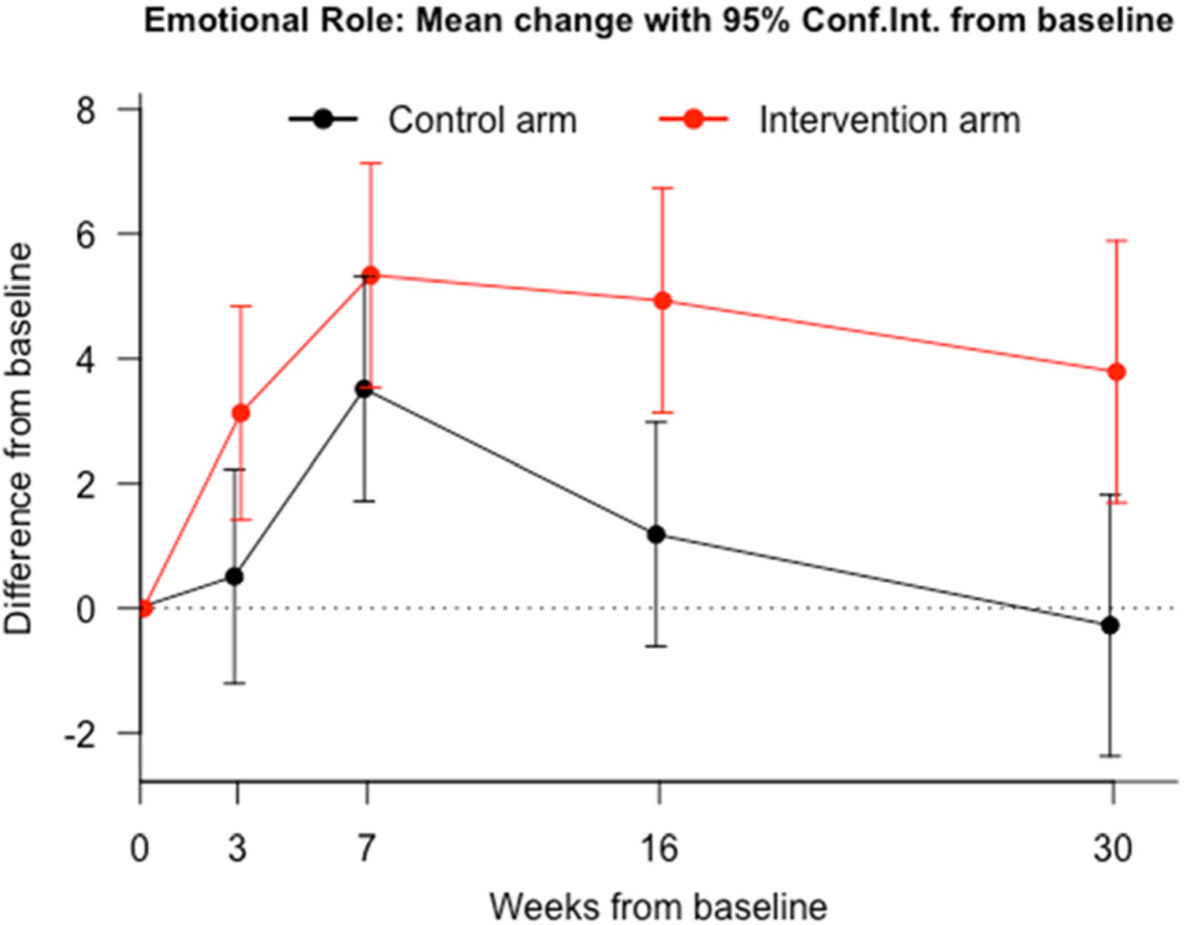
Evolution of emotional role functioning throughout the study in both groups. Comparison of means at baseline (A0), post-treatment at 3 weeks after baseline (A1), at 30 days follow-up after A1 (A2), at 3 months follow-up (A3), and at 6 months follow-up (A4)

Table 2 shows the effect of the group interventions, at each time point, by the terms of differences between baseline (A0) and each of the other 4 follow-up assessments (A1 to A4) of every SF-36v2 dimension. For both groups, SF-36v2 mean values increased in all dimensions in every time point. Significant differences (*p* < 0.05) were found, in favor of DDN group for all dimensions at the last follow up visit. However, for some dimensions such as PF (Fig. 2), PR (Fig. 3), SF and VT the evidence is stronger and more consistent from the beginning. The other dimensions did not show significant differences in all the assessments (Figs. 4 and 5). The summaries SF-36 PCS showed stronger differences towards the end, while SF-36 MCS showed stronger differences at the first and last visits.

**Table 2:** Intervention Program Effects. *Results from regression adjusted by baseline HRQoL centred in the mean*

Health related quality of life (SF-36)	^a^Control group (*n* = 64) Mean(SE)	^a^DDN group (*n* = 64) Mean(SE)	^b^Mean diff.	95% Confidence Interval	*P*-value
Physical Function					
A1 - A0	−4.36 (1.91)	6.54 (1.91)	10.9	(5.5 to 16.3)	0.00011
A2 - A0	0.52 (1.36)	7.44 (1.36)	6.92	(3.08 to 10.76)	0.00052
A3 - A0	−3.5 (2.01)	8.45 (2.01)	11.95	(6.28 to 17.62)	0.00006
A4 - A0	−4.19 (1.84)	7.64 (1.84)	11.83	(6.62 to 17.04)	0.00002
Physical Role					
A1 - A0	0.64 (0.9)	7.95 (0.9)	7.31	(4.8 to 9.82)	0.00000
A2 - A0	2.76 (1.02)	8.57 (1.02)	5.81	(2.95 to 8.68)	0.00010
A3 - A0	1.46 (1.28)	9.87 (1.28)	8.41	(4.82 to 11.99)	0.00001
A4 - A0	0.75 (1.1)	8.33 (1.1)	7.58	(4.51 to 10.65)	0.00000
Bodily Pain					
A1 - A0	5.59 (2.55)	12.81 (2.55)	7.22	(−0.08 to 14.52)	0.05241
A2 - A0	6.97 (2.38)	13.08 (2.38)	6.11	(−0.72 to 12.94)	0.07890
A3 - A0	−1.11 (2.95)	11.52 (2.95)	12.63	(4.2 to 21.07)	0.00364
A4 - A0	4.54 (2.76)	13.31 (2.76)	8.77	(0.86 to 16.69)	0.03012
General Health					
A1 - A0	−0.82 (1.44)	4.16 (1.44)	4.98	(0.93 to 9.02)	0.01644
A2 - A0	−1.3 (1.57)	2.58 (1.57)	3.87	(−0.52 to 8.27)	0.08381
A3 - A0	1.55 (1.74)	5.46 (1.74)	3.91	(−0.97 to 8.79)	0.11564
A4 - A0	−2.27 (1.58)	3.77 (1.58)	6.04	(1.62 to 10.46)	0.00783
Vitality					
A1 - A0	4.96 (1.96)	15.58 (1.96)	10.61	(5.11 to 16.12)	0.00021
A2 - A0	2.94 (1.91)	12.07 (1.91)	9.13	(3.77 to 14.49)	0.00099
A3 - A0	5.89 (2.13)	15.43 (2.13)	9.53	(3.56 to 15.51)	0.00199
A4 - A0	−0.91 (2.27)	16.28 (2.27)	17.19	(10.84 to 23.55)	0.00000
Social Function					
A1 - A0	−3.75 (1.89)	8.83 (1.89)	12.58	(7.3 to 17.86)	0.00001
A2 - A0	0.33 (1.96)	9.63 (1.96)	9.31	(3.82 to 14.8)	0.00105
A3 - A0	3.41 (1.69)	9.09 (1.69)	5.69	(0.97 to 10.41)	0.01856
A4 - A0	−6.92 (2.2)	11.61 (2.2)	18.53	(12.37 to 24.69)	0.00000
Emotional Role					
A1 - A0	0.51 (0.87)	3.13 (0.87)	2.62	(0.17 to 5.07)	0.03654
A2 - A0	3.52 (0.92)	5.34 (0.92)	1.82	(−0.75 to 4.39)	0.16409
A3 - A0	1.19 (0.92)	4.93 (0.92)	3.75	(1.18 to 6.32)	0.00463
A4 - A0	−0.27 (1.07)	3.79 (1.07)	4.06	(1.06 to 7.06)	0.00831
Mental Health					
A1 - A0	1.43 (1.97)	10.81 (1.97)	9.38	(3.82 to 14.94)	0.00116
A2 - A0	4.63 (1.6)	6.15 (1.6)	1.53	(−3.25 to 6.3)	0.52731
A3 - A0	9.6 (1.49)	11.26 (1.49)	1.66	(−2.87 to 6.19)	0.46848
A4 - A0	5.71 (1.71)	11.05 (1.71)	5.34	(0.38 to 10.3)	0.03498
SF-36 PSC					
A1 - A0	1.61 (0.65)	2.25 (0.65)	0.64	(−1.17 to 2.46)	0.48401
A2 - A0	0.48 (0.65)	1.8 (0.65)	1.31	(−0.58 to 3.21)	0.17201
A3 - A0	−1.41 (0.86)	1.86 (0.86)	3.26	(0.69 to 5.84)	0.01358
A4 - A0	−1.22 (0.75)	1.92 (0.75)	3.14	(0.96 to 5.31)	0.00512
SF-36 MCS					
A1 - A0	0.3 (0.87)	4.25 (0.87)	3.95	(1.5 to 6.39)	0.00185
A2 - A0	1.71 (0.58)	3.1 (0.58)	1.38	(−0.36 to 3.12)	0.11917
A3 - A0	4.04 (0.66)	4.8 (0.66)	0.75	(−1.25 to 2.76)	0.45702
A4 - A0	0.59 (0.75)	4.83 (0.75)	4.24	(2.07 to 6.41)	0.00018

*SE* standard error; VX-V0 model for differences in the corresponding SF-36 dimension between visit 0 and visit X;

^a^Expected change of SF-36 score between visits in a participant with average score at baseline;

^b^Expected Mean Difference between groups; SF-36 PSC: Physical Component Summary; SF-36 MSC: Mental Component Summary

## Discussion

This randomized controlled trial is the first that relates chronic non-specific neck pain, DDN of MTrPs and HRQoL. The results show that a Physical Therapy intervention with DDN plus stretching improved HRQoL, especially at PF, PR, SF and VT dimensions, in patients with chronic non-specific neck pain. The mentioned dimensions, describe physical activities limitation (PF), role limitations due to physical problems (PR), physical and emotional health problems interference in social life (SF), and vitality or tiredness feelings (VT). It means that, despite neck pain, participants perceived an improvement in their limitations after the proposed intervention and this perception lasted over time.

In the literature, there are many Physical Therapy interventions that are performed to improve symptoms of chronic non-specific neck pain, such as, pain intensity, mechanical hyperalgesia, neck range of motion, neck muscle strength and neck disability [4, 7–15, 20–24, 31, 32]. The combination of DDN and stretching has also shown effectiveness on improving the aforementioned symptoms [4, 20–24, 31, 32], as either DDN [4, 20–24, 31, 32] and stretching [8, 9, 14] have proved to be a good election on treating subjects with non-specific neck pain. Some studies report HRQoL in patients with chronic non-specific neck pain using different physical therapy interventions [8, 25–27], although they do not relate symptoms, DDN and HRQoL. Therefore, in this manuscript, the need of reporting HRQoL is important because it could not be performed in the primary analysis for the extension of the data.

HRQoL was measured with SF-36v2. As far as the authors know, currently there is no specific instrument to measure chronic non-specific neck pain, therefore most studies use SF-36 to report the effects of Physical Therapy interventions for improving HRQoL [33–35].

Regarding baseline values, the average age of participants is around 50 years old, most of them are women and overweight, similar to other studies´ samples on non-specific neck pain [8, 20, 25, 36]. Most SF-36 basal values obtained in both groups were lower than Spanish population reference values, especially for PR, BP and ER [37]. Some authors have established reduction of HRQoL 6 months after patients are diagnosed with chronic cervical pain. The participants of the present study had chronic nonspecific neck pain, and clinical data are representative of what occurs with neck pain subjects in developed countries and similar to those for other studies [38–40]. There were some remarkable SF-36 differences in baseline values between DDN and CG for PF, PCS and especially for BP, but these differences did not affect the results as the analysis by baseline values have been adjusted. The BP dimension of SF-36 assesses bodily pain in general, including other kinds of pain that the participants might suffer and cannot be mitigated by this intervention, so it might not be the most specific outcome to measure the effect of this intervention. It is common that people with chronic non-specific neck pain and other musculoskeletal alterations perceive worse health status and have limitations in their work or other daily life activities and this is reflected in their GH and ER dimensions.

In our data, the differences in the ER dimension were more significant in the later visits. This fact supports the idea that the negative impact of pain on HRQoL seems to be more related to the duration and sensation of limitation, when performing the daily life activities, than of its severity. It is also deeply related to functional, psychologic, social and working alterations [6, 33, 41]. The results show minimum clinically important differences [33, 35, 42] in some dimensions, which suggests that the inactivation of MTrPs could improve HRQoL in subjects with chronic non-specific neck pain.

Tekin et al. [32] performed a randomized double blinded clinical trial (*n* = 46) to assess the efficacy of DDN in patients with MPS, not only with chronic non-specific neck pain. They found similar results to the present study on pain and HRQoL, as all SF-36 dimensions values increased significantly in the group treated with DDN, while only VT dimension increased in the sham-placebo-superficial needling intervention group.

Cunha et al. [8] performed a randomized clinical trial (*n* = 33) comparing two groups where patients were treated during 1 h, twice a week during 6 weeks. One group intervention was 30 min of manual therapy and 30 min of stretching of upper trapezius, suboccipitalis, back of the neck, pectoralis major and minor, rhomboids, finger and wrist flexors, forearm pronators, finger and wrist extensors, forearm supinators, and paravertebral muscles. The other group received 30 min of manual therapy and 30 min of stretched muscle chains. They found that both groups reported improvement in all dimensions of SF-36 and concluded that this fact was probably due to the duration of the interventions, which could be enough time to influence the perception of the participant over the therapy received. In the present study, the intervention group obtained better results than control group in some dimensions of SF-36 (PF, PR, SF and VT) from the beginning and in all dimensions at the last follow up assessment. Therefore, the good results obtained in the present study are probably not related to patient’s perception.

Ris et al. [26] described the effectiveness of a physical training intervention plus specific exercises and pain education on HRQoL in subjects (*n* = 200) with chronic neck pain, during a 4-month follow up. The intervention group received exercises for neck/shoulder, balance and oculomotor function, graded physical activity training and pain education; CG received only pain education. They observed statistically significant differences in PCS and MCS SF-36 dimensions in favor of the intervention group.

In the present study, no endurance, or strengthening programs were performed. Inspite of that, an improvement of HRQoL was obtained which might reinforce the idea of the inactivation of MTrPs as the best way to improve HRQoL, otherwise, it could be a perpetuator factor maintaining the vicious cycle of the MTrP.

Participants’ therapeutic adherence and level of response in the questionnaires during assessments were high, similar to other study developed by Salo et al. [27]. This fact may be due to the attention and monitoring carried out by the researchers and that the questionnaires were completed in the researchers´ physical presence during the physical therapy assessments, while in most HRQoL studies the questionnaires were sent by mail or telephone. Other authors do not discuss this issue in their publications. In fact, in other studies some rehabilitative interventions were self-administered by participants, which may adversely influence results and therapeutic adherence [26, 43].

The authors consider that the present study has some limitations. Although consensus meetings were carried out before the study started and the same material was used for the educational intervention, passive stretching was performed by 2 different physical therapists which may have influenced the outcomes. Furthermore, the sample was composed of participants from just one health area which could bias the study’s external validity. The fact that this manuscript reports a secondary analysis might be a limitation. However, after reporting the primary results, the authors considered it necessary to develop a secondary analysis in order to describe the distinct effects of the intervention on HRQoL.

## Conclusions

Deep dry needling plus stretching is more effective than stretching alone for HRQoL improvement in people with non-specific neck pain, especially at long term. The evidence was stronger for PF, PR, SF and VT dimensions.

Future studies should strive to use high-quality condition-specific patient reported outcome instruments to determine the impact of special conditions and its physical therapy interventions on chronic non-specific neck pain subjects.

## Abbreviations

A0: Assessment at baseline
A2: Assessment at 1 month (A2–7 weeks from baseline)
A3: Assessment at 3 months (A3–16 weeks from baseline)
A3: Assessment just after the intervention period (3 weeks from baseline)
A4: Assessment at 6 months (A4–30 weeks from baseline)
BP: Bodily Pain
CG: Control group
DDN: Deep dry needling
ER: Emotional Role
GH: General Health
HRQoL: Health-related quality of life
MH: Mental Health
MPS: Myofascial pain syndrome
MTrPs: Myofascial trigger points
PF: Physical Function
PR: Physical Role
Pt1: Physical therapist 1
Pt2: Physical therapist 2
SF: Social Function
SF-36 MSC: Mental Component Summary
SF-36 PSC: Physical Component Summary
SF-36v2: Short Form 36 Health Survey Spanish version 2
VT: Vitality
